# Functional and Structural Outcomes of Temporal Zone II-Sparing Laser Photocoagulation Combined with Intravitreal Bevacizumab in Retinopathy of Prematurity

**DOI:** 10.1155/2018/2376240

**Published:** 2018-09-27

**Authors:** Eoi Jong Seo, Joo Yong Lee

**Affiliations:** Department of Ophthalmology, Asan Medical Center, University of Ulsan College of Medicine, Seoul, Republic of Korea

## Abstract

**Background/Aim:**

The aim of the study was to investigate the outcomes of temporal Zone II-sparing laser photocoagulation combined with intravitreal bevacizumab (IVB) in patients with Type 1 retinopathy of prematurity (ROP) in Zone I.

**Methods:**

Medical records of 74 eyes of 37 infants were analysed. Only infants with Type 1 ROP in Zone I were included. Thirty-two eyes were treated with temporal-sparing laser + IVB. Both Zone I and temporal Zone II were spared to minimise potential visual field loss. Forty-two eyes were treated with laser alone conventionally. Early treatment outcomes, late complications, and refractive errors were analysed.

**Results:**

The mean gestational age and birth weight of the enrolled patients were 25.7 ± 2.5 weeks and 798.8 ± 440.2 g, respectively. In the combined treatment group, plus sign regression was achieved faster (12.1 ± 6.2 days vs. 25.6 ± 21.3 days, *p*=0.011) and retreatment was required less (0% vs. 23.8%, *p*=0.004) than in the laser-alone group. Retinal/preretinal haemorrhages occurred more often in the laser-alone group (42.9% vs. 9.4%, *p*=0.002). Normal development of temporal retinal vessels was also observed in twelve eyes in the combined treatment group. No differences in late complications or refractive errors were observed between the groups.

**Conclusion:**

Temporal Zone II-sparing laser treatment combined with IVB showed good early treatment outcome and temporal retinal vessels development.

## 1. Introduction

Retinopathy of prematurity (ROP), a vasoproliferative disease associated with underdevelopment of the retina, is a leading cause of severe impairment of visual function in childhood [1]. Mild cases show spontaneous regression. However, some cases progress to a severe form of the disease and show retinal haemorrhage and tractional retinal detachment, which require surgical interventions such as vitrectomy [2, 3]. The Early Treatment for Retinopathy of Prematurity Cooperative Group (ETROP) trial established laser photocoagulation as an effective approach to stop disease progression in Type 1 ROP [4].

However, confluent laser scars on the retinal periphery may cause visual field restriction after the treatment. The area where laser photocoagulation was performed becomes atrophic, eventually restricting the patient's peripheral visual field. Several efforts have been made to reduce the area of laser photocoagulation using the recently highlighted intravitreal bevacizumab (IVB) treatment [5–8]. But IVB monotreatment showed delayed reactivation and caused devastating retinal sequelae in some cases [9]. Recent studies reported that posterior pole-sparing laser photocoagulation combined with IVB produced favourable outcomes [6, 7, 10, 11].

To minimise the visual field restriction caused by laser treatment, we saved a larger area in Type 1 ROP, including temporal Zone II, to maximise the combination effect compared with that in previously reported studies, which spared Zone I or the posterior pole. The functional and long-term structural outcomes were analysed.

## 2. Materials and Methods

This study, performed retrospectively, followed the tenets of the Helsinki declaration and was approved by the Institutional Review Board of Asan Medical Center. Informed consent was obtained from all parents after providing an explanation of laser photocoagulation and off-label use of bevacizumab, if indicated. Potential risks and side effects of IVB were explained. After thorough consideration, all parents agreed to the treatment involving laser photocoagulation with/without IVB.

The medical records of preterm infants who received laser photocoagulation alone or combined with IVB, and had been followed for at least 6 months in the period from Mar 2013 to Sep 2016 at Asan Medical Center, Seoul, Korea, were collected. Peripheral retinal laser photocoagulation was indicated when the fundus examination revealed Type 1 ROP in Zone I. Only infants with ROP in Zone I were included in the current study. Infants who received laser treatment due to Zone II ROP were excluded.

**Figure 1:**
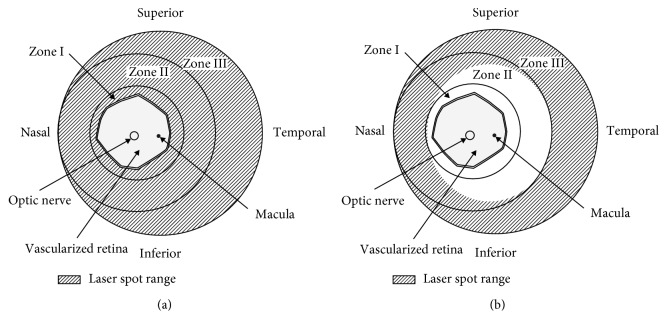
Diagrammatic images of the two different treatment methods. In conventional laser photocoagulation, laser spots covered the entire avascular area, including Zones I, II, and III (a). When both Zone I and temporal Zone II areas were spared, intravitreal bevacizumab was added (b). Slash lines indicate the laser spot range.

Two distinctive patterns of treatment were performed (Figure 1). In one group, eyes were treated with laser photocoagulation only. Laser photocoagulation was done using an 810 nm laser indirect ophthalmoscope (LIO). In these cases, LIO treatment was performed conventionally in the entire avascular area from the retinal ridge to the ora serrata. These patients were defined as the LIO-alone group. In the other group, patients were treated with both LIO and IVB (0.5 mg/0.02 mL). When combined with IVB, LIO treatment spared Zone I and temporal Zone II to minimise potential visual field loss. These patients were classified as the temporal-sparing LIO + IVB group. The treatment choice was determined by a retinal specialist (JYL) depending on the severity of ROP. In cases of aggressive posterior ROP (AP-ROP), poor pupillary dilation, or media opacity, combined laser treatment with IVB was performed instead of laser-only treatment.

Before treatment and during follow-ups, the fundus was examined by indirect ophthalmoscopy and wide-field photography, if indicated. A RetCam (Clarity Medical Systems, Pleasanton, CA, USA) was employed to record the severity of ROP, extent of the laser scar, regression of new vessels, and development of peripheral retinal vessels after treatment. Patient baseline characteristics such as gestational age at birth, birth weight, multiplicity, APGAR score, ventilator care, and O_2_ therapy period were collected. A series of comorbid systemic diseases associated with ROP, such as necrotising enterocolitis, respiratory distress syndrome, ligated patent ductus arteriosus, bronchopulmonary dysplasia, sepsis, intrauterine growth restriction, poor weight gain, hydrocephalus, and intraventricular haemorrhage, were also documented.

Early treatment outcomes/complications as well as late structural/functional complications were analysed based on fundus photographs and medical records. At 6–12 months after treatment, all treated infants underwent measurement of refractive errors by manual cycloplegic refraction performed by a skilled paediatric ophthalmologist. The results were recorded as measurements of spherical and cylinder astigmatism, and the spherical equivalent. These were also divided into the categories of emmetropia, mild (0–3 D), moderate (3–6 D), or high (>6 D) myopia, and hyperopia.

Statistical analysis was performed using SPSS for Windows (version 21.0, SPSS, Inc., Chicago, IL). The independent *t*-, Fisher's exact, and Pearson's Chi-square tests were used. *p* values < 0.05 were considered to be statistically significant.

## 3. Results

A total of 74 eyes of 37 infants were analysed. Thirty-two eyes of 16 infants were treated with temporal-sparing LIO + IVB, and 42 eyes of 21 infants were treated with LIO alone. The mean follow-up period was 19.7 ± 9.0 months. Out of the 32 eyes treated with temporal-sparing LIO + IVB, 10 (31.3%) showed AP-ROP. With the exception of the follow-up period being shorter in the temporal-sparing LIO + IVB group, there were no statistically significant differences in baseline characteristics and comorbid conditions between the two groups (Table 1).

Treatment outcomes and complications are shown in Table 2. After both treatments, all eyes showed complete regression of plus sign. However, plus sign regression was faster in the group combining LIO treatment with IVB compared with the LIO-only group (12.1 ± 6.2 vs. 25.6 ± 21.3 days, *p*=0.011). Ten eyes in the LIO-alone group required retreatment after a mean 25 days from the initial treatment, while all eyes in the temporal-sparing LIO + IVB group were stabilised after the first treatment (*p*=0.004). Among the eyes requiring retreatment, 6 eyes were treated with LIO + IVB and 4 eyes were treated with additional LIO only. Retinal/preretinal haemorrhages were found more frequently in the LIO-alone group (9.4% vs. 42.9%, *p*=0.002). No reports of endophthalmitis or adverse systemic safety issues related to IVB were found. Among late complications, structural complications like macular dragging or optic disc atrophy tended to occur frequently in patients treated with LIO alone. Functional complications such as strabismus or nystagmus showed no tendency between the groups. Refractive error measurements were performed 10.4 ± 5.0 months after treatment. No statistically significant differences were found between the two groups in the spherical equivalent (*p*=0.293) and refractive error (*p*=0.130) distributions.

**Figure 2:**
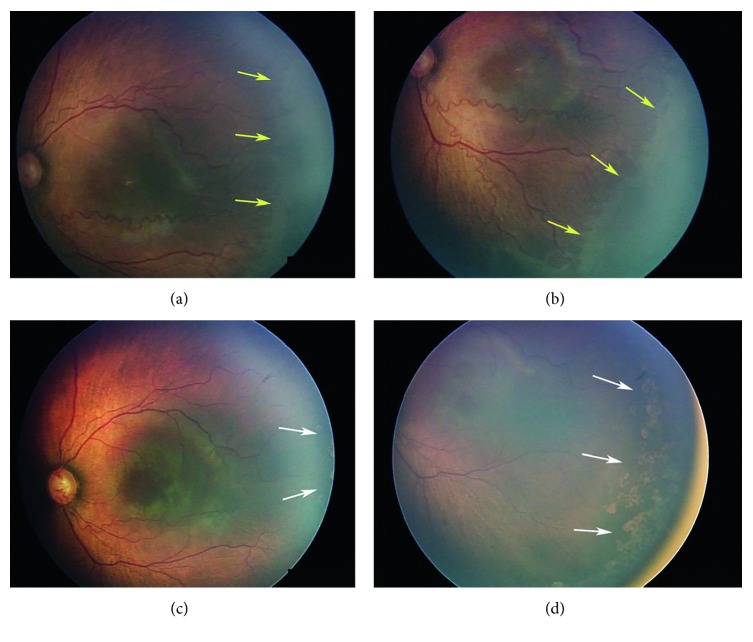
A male infant born at a gestational age of 27 weeks showed Type 1 ROP at 32 weeks PMA. (a, b) An avascular area was observed and is indicated with yellow arrows. Temporal-sparing LIO combined with IVB was performed. Two weeks after treatment, temporal retinal vessels had reached the laser-treated Zone II area. (c, d) The edge of the vascularized retina is indicated with white arrows. Reactivation of ROP was not observed through 137 weeks of follow-up.

**Figure 3:**
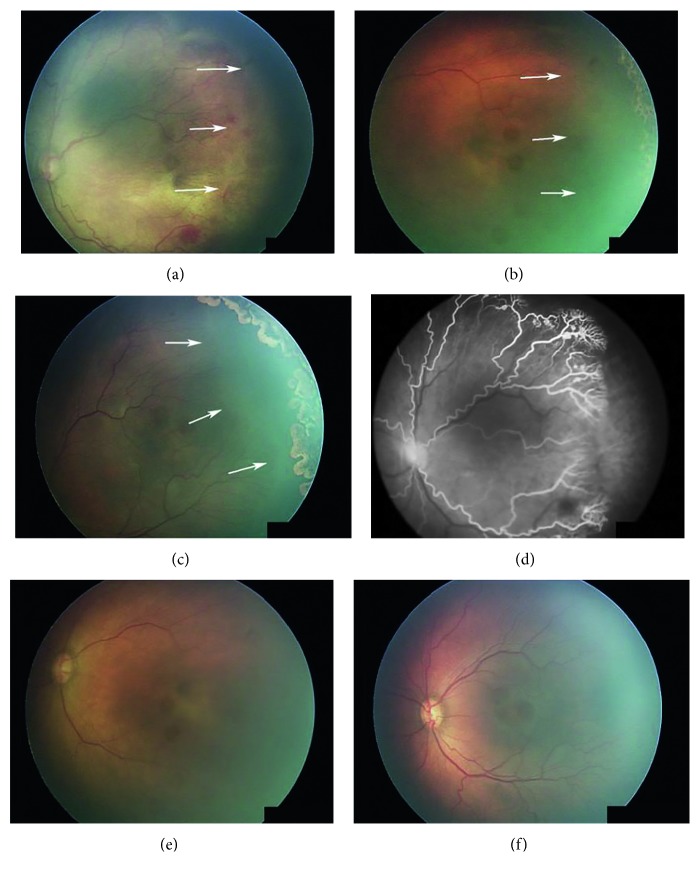
Examples of eyes with persistent avascular areas after temporal-sparing LIO + IVB. Avascular areas are indicated with white arrows. (a, d) Pretreatment fundus image and fluorescein angiograph. (b, e) Two weeks after treatment, the plus sign had disappeared but the temporal avascular area persisted. (c, f) Three months after treatment, plus sign was absent but the temporal avascular area had not decreased compared with the previous exam.

Among the 32 eyes treated with temporal-sparing LIO + IVB, 24 eyes of 12 infants were able to analyse the retinal periphery with wide-field fundus photography after 1 month of treatment. The others were not able to do so because of poor peripheral visualisation. Normal development of temporal retinal vessels was observed in the 12 eyes (50%) of 6 infants. Vessel development progressed close to the laser scar, and the avascular area was barely visible (Figure 2). In the other 12 eyes, no or little vessel development was observed, and most of the avascular area remained. Among these, 5 eyes (both eyes of 2 infants, the left eye of 1 infant) showed a persistent avascular area even 3 months after treatment (Figure 3). No significant correlation between the presence of a persistent avascular area and any other patient characteristic was found.

## 4. Discussion

In the present study, the functional and structural outcomes in patients with Type 1 ROP were compared between those who underwent temporal Zone II-sparing laser photocoagulation combined with IVB and conventional laser photocoagulation alone. Wider preservation of the retina after regression of ROP was observed in eyes treated with temporal-sparing laser therapy combined with IVB compared with those treated with laser photocoagulation alone. Eyes undergoing the combined treatment required retreatment less frequently and showed faster regression of plus sign compared with eyes treated with LIO alone. Refractive changes and the incidence of long-term complications showed no differences between the two treatments. In 50% of eyes treated with the combined therapy, normal development of the peripheral retina was observed after complete regression of ROP.

Temporal Zone II area was chosen to be spared in the current study. Saving the nasal area would be more beneficial in saving the temporal visual field in binocular vision. However, as shown in Figure 1, the temporal area is larger, and it is mainly responsible for the retinal avascularity and has spatial closeness with the macula. Saving the maximum amount of retina adjacent to the macula could enhance the central visual field, which is crucial for the functional vision.

Because retinal atrophy and photoreceptor damage are inherent sequelae of laser photocoagulation, IVB monotherapy might be a good option to achieve ROP regression with a good anatomical outcome. However, several findings of delayed reactivation were reported in IVB monotherapy, sometimes as late as 3 years posttreatment [9, 12–14]. Moreover, when recurrences occur, their locations and patterns may be altered compared with the original pathology [9, 13, 15]. Several efforts have been made to combine laser photocoagulation and IVB to minimise retinal damage and maximise treatment efficacy, including studies combining IVB therapy with Zone I or posterior pole-sparing laser treatment [10, 16, 17]. We performed eccentric laser photocoagulation on the avascular area sparing the temporal Zone II area and injected bevacizumab simultaneously. While maximising the effect of combined IVB and laser therapy, we were able to save a larger area of the retina compared with the previously published reports, in which only Zone I or the posterior pole was saved. The long-term outcomes of our treatment were not different from those of conventional laser therapy alone, while better visual function, such as wider visual field acquisition, can be expected.

As in previous reports [5, 7, 8], combining IVB and laser therapy in one treatment produced better structural outcomes than did laser treatment alone. In the present study, plus sign regression was faster and retreatment was required less frequently in the combined therapy group compared with the laser-only group (Table 2). Based on the pathogenesis of ROP, a surge of VEGF excreted from the wide avascular retina plays a key role in neovascularisation [18, 19]. The rapid VEGF-lowering effect of injected bevacizumab is beneficial for stabilisation of the retinal vasculature. The diminished laser ablation of the retina in combination therapy resulted in fewer retinal/preretinal haemorrhages (Table 2). Given that the ocular environment is proangiogenic and proinflammatory, fibrin-rich preretinal haemorrhage can be a possible risk for fibrovascular membrane formation [20]. The rapid stabilisation of ROP achieved with IVB may underlie the decrease in structural complications in long-term follow-up. In the present study, macular dragging was more frequently observed in the LIO-alone group with a marginal significance (*p*=0.065).

In spite of the advantages of IVB use in ROP treatment, delayed reactivation is a major concern. However, there was no reactivation in both groups followed up for 103.1 ± 43.6 weeks PMA in the current study. The follow-up period was shorter in the combined treatment group (84.4 ± 36.9 vs. 117.4 ± 43.6 weeks). Nonetheless, given that the mean time to recurrence was 19.2 ± 8.6 weeks after bevacizumab treatment in the BEAT-ROP study, our follow-up period was long enough to analyse long-term efficacy [8]. Another case series [9] reported reactivation of ROP after IVB monotherapy as late as at 69 weeks PMA. When IVB was combined with Zone I-sparing laser treatment, neither reactivation nor retinal detachment was reported for 83.6 weeks PMA [21]. Treatment of ROP with bevacizumab followed by prophylactic laser therapy also showed no reactivation for 125 weeks PMA [17]. Taken together with our findings, these results demonstrate that combining laser therapy with IVB treatment is a safe way to prevent delayed reactivation of ROP.

After treatment, there was no difference in mean spherical equivalent between the two groups at 6 months. Over 80% of the infants showed emmetropia or mild myopia. In a meta-analysis of 11 studies of a total of 378 eyes with intravitreal anti-VEGF treatment in ROP, the average spherical equivalent refractive error reported after anti-VEGF monotherapy ranged from +0.75 D to −3.75 D [22]. In the BEAT-ROP study, ROP-severity-matched eyes receiving anti-VEGF therapy were found to have significantly lower myopia than those receiving peripheral laser ablation at average of 2.5 years [23]. Further observation of refractive errors should be required to obtain refractive error differences.

As demonstrated in Figure 2, 12 out of 24 eyes treated with IVB showed normal development of the peripheral retina in this study. Peripheral retinal development after IVB treatment was reported in multiple previous studies [5, 6, 17, 24, 25]. Lepore et al. reported various abnormalities such as abnormal branching or shunts at the retinal periphery of bevacizumab-treated eyes [25]. It is unclear whether these peripheral vessels act as normal vasculature, leading to normal peripheral retinal development. However, because ablation with laser certainly destroys the retinal structure, preservation of as much viable retina as possible should be good for the patient's potential visual field. Five eyes (20.8%) in the current study showed persistent avascular areas 3 months after temporal-sparing laser treatment combined with IVB. If it persists further, the avascular area can be worrisome because it can be a source of VEGF that can potentially lead to the late reactivation of ROP. Although there were no infants with late reactivation of ROP in the present study, a longer study is needed to identify the possibility of using additional laser treatment for the persistent avascular areas.

There have been reports that performing laser treatment in combination with bevacizumab injection increases the systemic levels of the drug, which decreases the levels of systemic VEGF and could possibly be associated with decreased neurodevelopmental outcomes [26, 27]. The use of laser can alter the blood retinal barrier, potentially increasing the anti-VEGF absorption into the systemic flow. Within the follow-up period of our study, no neurodevelopmental delay related to IVB was observed. Further studies would be helpful in revealing the influence of IVB on the systemic VEGF.

Our study has several limitations. Besides the retrospective nature of the study design and small sample size, the major limitation is that multiple systemic and environmental factors influencing the treatment outcome, such as oxygen supply, nutrient concentration, intrauterine environment, and perinatal issues, may have been different between the treatment groups. We confirmed that major systemic risks were not significantly different between the two groups in Table 1, but other factors could still play confounding roles. Another limitation is the possibility of a selection bias due to the treatment method being chosen by a single ophthalmologist. However, as mentioned above and shown in Table 1, the more severely affected eyes tended to be treated with temporal-sparing LIO + IVB. Finally, we could not measure peripheral visual function as a treatment outcome. Because subjects were infants, the conventional visual field test could not be performed. Retinal microperimetry could be used instead to clarify the functional outcome of the peripheral retina. When the infants are grown enough to undergo the visual field test, a quantitative comparison can be performed.

Despite its limitations, the present study is valuable in that we demonstrated good structural and functional outcomes in minimally threatened visual fields and visualised them with wide-field fundus photography and fluorescein angiography. The previous studies had limitations in treatment standardisation or diagnosis because of a lack of images. We adopted a wide-field fundus-imaging system to determine the ROP stage, plus sign regression, retinal haemorrhages, extent of the avascular area, laser ablated area, and peripheral vessel advancement. This visualisation allowed us to standardise treatment and perform an appropriate comparison.

## 5. Conclusions

Temporal-sparing laser treatment combined with intravitreal bevacizumab showed better early treatment outcomes compared with conventional laser treatment. There were no differences between the groups in long-term structural and functional complications, including delayed reactivation. Because of the preservation of the viable retina with peripheral vessel development and the low reactivation rate, temporal Zone II-sparing laser photocoagulation combined with intravitreal bevacizumab could be a good choice for Type 1 ROP patients to achieve complete regression while minimising peripheral field defects.

## Figures and Tables

**Table 1:** Baseline characteristics of the subjects.

	Temporal-sparing LIO + IVB	LIO alone	*p* value
Number of infants (eyes)	16 (32)	21 (42)	
Gestational age at birth (weeks)	25.1 ± 1.3	26.2 ± 3.0	0.169^‡^
Birth weight (g)	673.1 ± 145.8	594.5 ± 557.3	0.132^‡^
Follow-up period (weeks)	84.4 ± 36.9	117.4 ± 43.6	0.020^‡^
Eyes with aggressive posterior ROP (%)	10 (31.3)	0 (0)	<0.001^*∗*^
Multiplicity (%)			0.502^*∗*^
Single	9 (56.3)	12 (57.1)	
Twins	6 (37.5)	9 (42.9)	
Triplets	1 (6.2)	0 (0.0)	
APGAR score			
1 minute	3.1 ± 2.2	4.0 ± 1.6	0.186^‡^
5 minutes	5.2 ± 2.7	6.1 ± 1.1	0.155^‡^
Ventilator care period (days)	75.6 ± 29.0	67.1 ± 35.0	0.427^‡^
O2 therapy period (days)	96.9 ± 30.3	88.4 ± 42.9	0.485^‡^
Comorbid conditions			
NEC operation (%)	5 (31.3)	2 (9.5)	0.095^*∗*^
PDA ligation (%)	12 (75.0)	20 (95.2)	0.144^†^
RDS (%)	15 (93.8)	21 (100)	0.432^†^
BPD ≥ moderate (%)	5 (31.3)	12 (57.1)	0.117^†^
Sepsis (%)	11 (68.8)	12 (57.1)	0.471^*∗*^
IUGR (%)	2 (12.5)	7 (33.3)	0.248^†^
Poor weight gain (%)	15 (93.8)	16 (76.2)	0.206^†^
Hydrocephalus (%)	2 (12.5)	7 (33.3)	0.248^†^
IVH (%)			0.448^*∗*^
None	4	2	
Grade I-II	9	14	
Grade III-IV	3	5	

Continuous variables are shown as mean ± SD; ^*∗*^chi-square analysis; ^†^Fisher's exact test; ^‡^Student's *t*-test; NEC, necrotising enterocolitis; PDA, patent ductus arteriosus; RDS, respiratory distress syndrome; BPD, bronchopulmonary dysplasia; IUGR, intrauterine growth restriction; IVH, intraventricular haemorrhage.

**Table 2:** Comparison of treatment outcomes, ocular complications, and refractive errors.

	Temporal-sparing LIO + IVB	LIO alone	*p* value
Early treatment outcome			
Plus sign disappeared (%)	32 (100)	42 (100)	
Time to plus sign regression (days)	12.1 ± 6.2	25.6 ± 21.3	0.011^‡^
Retreatment (%)	0 (0)	10 (23.8)	0.004^†^
Time to retreatment (days)		25.0 ± 14.4	
Retinal/preretinal hmr (%)	3 (9.4)	18 (42.9)	0.002^*∗*^
Vitreous hmr (%)	0 (0)	4 (9.5)	0.129^†^
Ocular inflammation (%)	0 (0)	0 (0)	
Endophthalmitis (%)	0 (0)	0 (0)	
Adverse systemic safety issues (%)	0 (0)	0 (0)	

Late complications			
Macular dragging (%)	0 (0)	5 (11.9%)	0.065^†^
Optic disc atrophy (%)	4 (12.5)	8 (19.0%)	0.449^*∗*^
Strabismus (%)	10 (31.3)	14 (33.3)	0.850^*∗*^
Nystagmus (%)	6 (18.8)	6 (14.3)	0.606^*∗*^
Amblyopia (%)	8 (25.0)	10 (23.8)	0.906^*∗*^
Cataract (%)	0 (0)	0 (0)	
Microcornea (%)	0 (0)	0 (0)	
Angle closure glaucoma (%)	0 (0)	0 (0)	

Refractive errors			
Measurement period (months)	9.7 ± 4.2	11.0 ± 5.6	0.464^‡^
Spherical equivalent (D)	−0.43 ± 0.58	−0.94 ± 2.69	0.293^‡^
Emmetropia (%)	7 (21.9)	16 (38.0)	0.130^*∗*^
Mild myopia (0–3 D) (%)	20 (62.5)	22 (52.4)	
Moderate myopia (3–6 D) (%)	0 (0)	0 (0)	
High myopia (>6 D) (%)	0 (0)	2 (4.8)	
Hyperopia (%)	5 (15.6)	2 (4.8)	

Continuous variables are shown as mean ± SD; ^*∗*^chi-square analysis; ^†^Fisher's exact test; ^‡^Student's *t*-test; hmr, haemorrhage.

## Data Availability

The data used to support the findings of this study are available from the corresponding author upon request.
